# Effects of marathon race on selected myokines and sclerostin in middle-aged male amateur runners

**DOI:** 10.1038/s41598-021-82288-z

**Published:** 2021-02-02

**Authors:** Ewa Śliwicka, Tomasz Cisoń, Łucja Pilaczyńska-Szcześniak, Andrzej Ziemba, Anna Straburzyńska-Lupa

**Affiliations:** 1 Department of Physiology and Biochemistry, Poznan University of Physical Education, Królowej Jadwigi Str. 27/39, 61-871 Poznań, Poland; 2 Department of Physiotherapy, State University of Applied Science in Nowy Sącz, Nowy Sącz, Poland; 3 Faculty of Rehabilitation and Sport, The President Stanisław Wojciechowski State University of Applied Sciences in Kalisz, Kalisz, Poland; 4 Department of Applied Physiology, Mossakowski Medical Research Centre, Polish Academy of Sciences, Warsaw, Poland; 5 Department of Physical Therapy and Sports Recovery, Poznan University of Physical Education, Poznań, Poland

**Keywords:** Cytokines, Hormones

## Abstract

In recent years, there has been increasing interest in the homeostatic response to extreme exercises, especially in the integrated function of muscle and bone. The aim of this study was to evaluate the effects of a marathon race on selected myokines and sclerostin in 10 male recreational runners (mean age 41 ± 7.7 years). Body composition, bone mineral density (BMD), and the serum concentration of myostatin, irisin, sclerostin, osteoprotegerin (OPG), 25-hydroxyvitamin D (25(OH)D), parathyroid hormone (PTH), high-sensitivity interleukin-6 (hsIL-6), tumor necrosis factor α (TNFα), high-sensitivity C-reactive protein (hsCRP) and myoglobin, were determined 24 h before and 24 h and 72 h after a marathon race. Post-marathon increases were observed in the levels of myostatin (1.2-fold), OPG (1.5-fold), and PTH (1.3-fold), hsIL-6 (1.9-fold), myoglobin (4.1-fold), hsCRP (fivefold), TNFα (2.6-fold), after 24 h; and in myostatin (1.2-fold), irisin (1.1-fold), sclerostin (1.3-fold), OPG (1.3-fold), and PTH (1.4-fold), hsIL-6 (1.4-fold), TNFα (1.9-fold), after 72 h compared to the baseline level. The results show that in response to the marathon run, a complex network of endocrine interactions is initiated. Further research is needed to fully elucidate the long-term impact of prolonged high intensity exercise on the human body.

## Introduction

In recent years, long-distance running has become increasingly popular among people of different ages^1^. Although physical exercise is an important component of a healthy lifestyle, still little is known how homeostatic response to long-lasting exercises performed during a marathon run influences muscle-bone crosstalk^2^.

A large component of long-distance running involves eccentric muscle contractions, which can lead to different levels of damage in the muscles and connective and bone tissue^3,4^. Exercise-induced muscle damage (EIMD) is associated with delayed-onset-muscle-soreness (DOMS), muscle weakness and a decrease range of motion^5^. EIMD has been associated with inflammatory response, which is crucial for the repair of damaged tissue^6^. The inflammatory cascade is characterized by an initial proinflammatory response (1.5–24 h after exercise) and anti-inflammatory muscle regenerative response (24–72 h after exercise)^6,7^.

Skeletal muscles and bones are closely related and play a key role in the physical health of humans^8^. In response to exercise, these organs communicate not only via mechanotransduction, but also through the endocrine system via myokines (e.g., myostatin, irisin, IL-6) and osteokines (e.g., sclerostin). In addition, it is necessary to take into account the effect of adipokines (e.g., TNF-α released from adipose tissue) on both these secretory organs^2^.

Myokines participate in the autocrine regulation of metabolism, angiogenesis, and muscle growth, as well as in the paracrine and endocrine regulation of other tissues and organs, such as bone, liver, brain, and adipose tissue^9^. Exercise-induced myokines can have an anti-inflammatory effect in acute inflammation (during the course of infection) and chronic low-grade inflammation (due to aging or metabolic disorders)^10^.

Myostatin and irisin play opposing roles in the functional bone-muscle unit^8^. Moreover, studies have indicated that both myokines have contrasting effects on muscle mass and strength via the IGF-1/Akt/mTOR pathway^11,12^. Namely, while myostatin reduces protein synthesis and increases protein degradation in skeletal muscles^11^, irisin is a pro-myogenic factor that induces skeletal muscle hypertrophy^12^.

Myostatin belongs to the transforming growth factor-β superfamily (TGF β). It is mainly expressed in skeletal muscle as negative regulator of its mass^2^. Myostatin has also negative impact on bone remodelling, enhancing catabolic and resorptive state trough increased osteoclastogenesis and reduced bone formation^2^.

Irisin is produced primarily by muscles and is released into the circulation during physical activity, resulting in an increased energy expenditure, oxidative metabolism, and improved glucose metabolism^13^. Studies on animal models showed that irisin can improve osteoblastogenesis and bone mass^14,15^.

The main source of IL-6 release into the circulating blood is the contracting skeletal muscle in response to exercise. IL-6 participates not only in inflammatory response, but also in glucose uptake, fatty acid oxidation and bone metabolism via increasing osteoclast formation and osteoblast differentiation^16^.

Sclerostin, glycoprotein mainly secreted by osteocytes, acts as an antagonist of bone formation through the canonical Wnt/β-catenin signaling pathway^17^. The Wnt/β-catenin signaling pathway plays a role in insulin resistance, inflammation, metabolic disturbance^17^, and skeletal muscle regeneration^18^. Recent studies indicate that sclerostin can also acts as a bone regulator^17,18^. Robling et al.^19^ demonstrated that sclerostin expression decreased by loading, leading to increased bone formation. These results suggesting that sclerostin may be a key protein involved in mechanical loading. Moreover, bone forming cells possess nuclear receptors for 1,25(OH)_2_D and membrane receptors for PTH^20^.

Vitamin D is a hormone that acts and integrates bone and muscle function. Its indirect action is related to calcium and phosphate levels, while direct effects is connected to local activity of the vitamin D receptor in some tissues (e.g., skeletal muscle, adipose tissue). Vitamin D may also indirectly affecting muscle-bone crosstalk via regulation of muscle and bone-derived hormones, like myostatin, IL-6 and sclerostin^21^.

PTH is a key hormone in the metabolism of calcium and phosphates, playing an important role in neuromuscular signaling, muscle contraction and the biosynthesis of adenosine triphosphate (ATP) and other energy substrates. Therefore, exercise can affect the expression and secretion of PTH^22^.

Although regular physical activity has benefit on muscle and bone health^23^, a scientific evidence indicates that long distance running can also have adverse effects^24,25^. Among others, it has been observed that men who run long distances (> 100 km per week) have a decreased bone mass and increased bone turnover compared to controls, which is indicative of an acceleration of bone loss^26^. Further research into the molecular physiology of physical exercise is need to better understand the mechanisms of exercise-induced health effects and prevent its potentially negative influence.

In this study, we investigated the impact of extreme exertion experienced during a marathon race on selected myokines and sclerostin in middle-aged male amateur runners during the Visegrad Marathon, one of the most difficult marathons in Europe due to changes in altitude.

## Results

**Figure 1:**
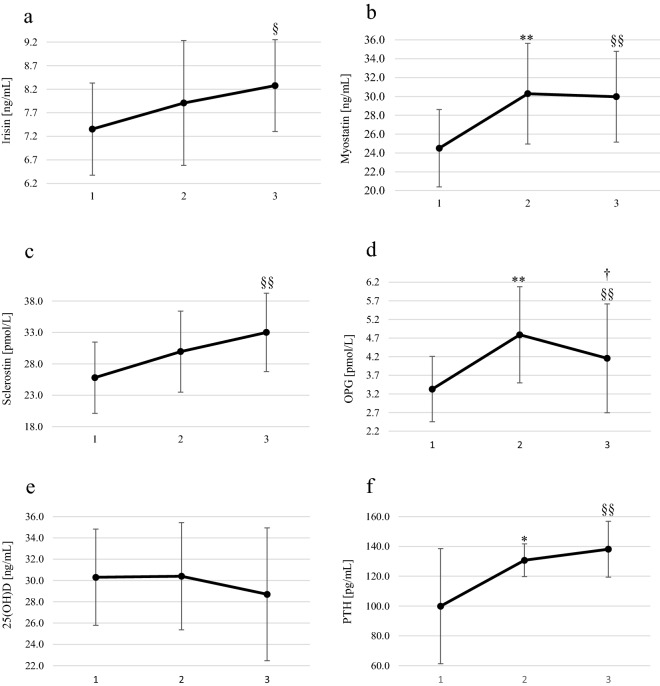
Blood concentrations of irisin, myostatin, sclerostin, OPG, 25(OH)D and PTH in male amateur runners before and after the Visegrad Marathon. (**a**) irisin, (**b**) myostatin, (**c**) sclerostin, (**d**) OPG, (**e**) 25(OH)D, (**f**) PTH. 1: before marathon; 2: 24 h after the marathon; 3: 72 h after the marathon. ** p < 0.01 significant differences between measurements before and 24 h after the marathon. * p < 0.01 significant differences between measurements before and 24 h after the marathon. † p < 0.05 significant differences between measurements 24 h and 72 h after the marathon. ^§§^ p < 0.01 significant differences between measurements before and 72 h after the marathon. ^§^ p < 0.05 significant differences between measurements before and 72 h after the marathon.

**Figure 2:**
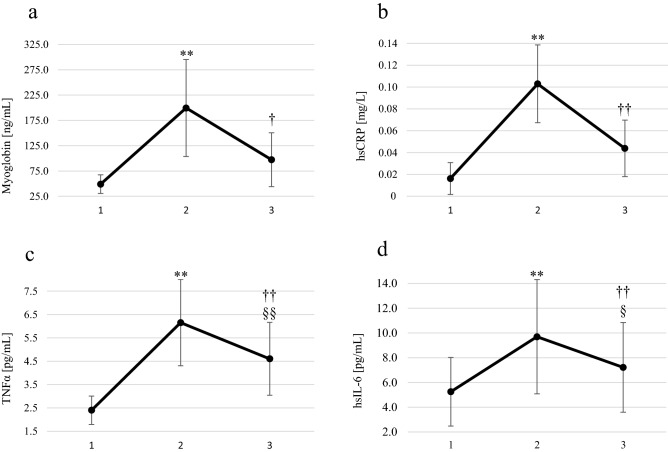
Blood concentrations of myoglobin, hsCRP, TNFα, and hsIL-6 in male amateur runners before and after the Visegrad Marathon. (**a**) myoglobin, (**b**) hsCRP, (**c**) TNFα, (**d**) hsIL-6. 1: before marathon; 2: 24 h after the marathon; 3: 72 h after the marathon. ** p < 0.01 significant differences between measurements before and 24 h after the marathon. †† p < 0.01 significant differences between measurements 24 h and 72 h after the marathon. † p < 0.05 significant differences between measurements 24 h and 72 h after the marathon. ^§§^ p < 0.01 significant differences between measurements before and 72 h after the marathon. ^§^ p < 0.05 significant differences between measurements before and 72 h after the marathon.

One day (24 h) after finishing the marathon, the blood levels of myostatin (1.2-fold), OPG (1.5-fold), and PTH (1.3-fold) (Fig. 1) as well as of increased of myoglobin (4.1-fold), hsCRP (fivefold), TNFα (2.6-fold) and hsIL-6 (1.9-fold) (Fig. 2), compared to the baseline level. After 72 h, the levels of myostatin (1.2-fold), irisin (1.1-fold), sclerostin (1.3-fold), OPG (1.3-fold), and PTH (1.4-fold) (Fig. 1) and also increased TNFα (1.9-fold) and hsIL-6 (1.4-fold) (Fig. 2) compared to the baseline level. In the preliminary study, we observed a significant correlation between TNF-α and myostatin (r = 0.64, *p* = 0.049).

As shown in Table 1, we found a positive correlation between changes (Δ_1-2_) in the concentration of myostatin and hsCRP, as well as sclerostin and hsIL-6, and a negative correlation between irisin and TNF-α. The correlation analysis of these changes (Δ_1-3_) indicated that sclerostin was positively correlated with hsIL-6. Similar correlations were observed for the 25(OH)D and irisin levels. A negative relationship was observed between sclerostin and myostatin, as well as PTH and OPG.

**Table 1:** Spearman's rank correlation coefficients of tested variables.

Variables	R	*p-*value
Δ_1-2_ Myostatin/Δ_1-2_ hsCRP	0.64	0.0479
Δ_1-2_ Irisin/Δ_1-2_ TNFα	− 0.79	0.0061
Δ_1-2_ Sclerostin/Δ_1-2_ hsIL-6	0.66	0.0376
Δ_1-3_ Irisin/Δ_1-3_ 25(OH)D	0.72	0.0190
Δ_1-3_ TNFα/Δ_1-3_ Myoglobin	0.79	0.0061
Δ_1-3_ Sclerostin/Δ_1-3_ hsIL-6	0.73	0.0158
Δ_1-3_ Sclerostin/ Δ_1-3_ Myostatin	− 0.66	0.0376
Δ_1-3_ PTH/ Δ_1-3_ OPG	− 0.82	0.0032

1 = before marathon race, 2 = 24 h after marathon race, 3 = 72 h after marathon race.

## Discussion

The main findings of our study clearly indicate that the marathon race contributed to a significant increase in the concentrations of myokines (myostatin, irisin, hsIL-6) and sclerostin. At the same time, changes in other biochemical parameters associated with the inflammatory response and muscle and bone metabolism were observed.

Irisin and myostatin are believed to be secreted from skeletal muscles inversely after physical activity^27^, wherein increasing the amount of irisin could inhibit myostatin synthesis^8,28^. Our results appear to confirm this relationship: the levels of irisin grew significantly, reaching their highest concentration at 72 h post-marathon compared to the baseline level, while a significant increase in myostatin was observed 24 h post- marathon (the highest value), and plateau at 72 h post-marathon.

Furthermore, when analyzing our baseline data, we identified a positive correlation between myostatin and TNFα, as well as a correlation between the changes (Δ_1-2_) in the concentration of myostatin and hsCRP. Our results suggest that myostatin has a negative effect on muscle tissue via its involvement in inflammation^29^.

Our results are in agreement with those of previous studies^29,30^, in which a significant increase in the myostatin levels was observed up to 48–72 h after a single bout of exercise. Several factors may influence the expression of myostatin, including the duration^31^ or intensity of the physical activity. Pereirea et al.^32^ used an animal model to show that overtraining leads to inflammation and the upregulation of myostatin.

Research conducted by Kraemer et al.^33^ showed that prolonged exercise moderate intensity on a treadmill leads to a temporary increase in the amount of circulating irisin, which occurs in the first hour of exercise and decreases after 90 min of exercise and 20 min of recovery. Some authors have pointed out that acute exercise transiently increases the circulating levels of irisin, while chronic exercise training does not cause changes or decreases in the basic level of irisin^33,34^.

Despite an increasing number of studies, the biological effects of irisin in humans remains largely unknown. Therefore, further research is needed to determine how irisin acts beyond 72 h post-exercise. According to Mazur-Biały et al.^35^, irisin has potential anti-inflammatory properties, which supports the negative correlation between the changes (Δ_1-2_) in irisin and TNFα observed in our study. High concentrations of irisin decreased the levels of pro-inflammatory cytokines by suppressing certain signaling pathways, resulting in a reduction of the levels of RANKL^35^.

Research carried out in recent years has demonstrated that sclerostin is important for bone homeostasis^2,36^, and its expression in osteocytes has been found to be negatively regulated by mechanical loading^37^.

We observed a significant increase in the levels of sclerostin only 72 h post-marathon compared to the baseline level. In contrast, other studies have reported a range of changes. For example, Kerschan-Schnidl et al.^29^ observed that the sclerostin levels did not change immediately or 72 h post-run in ultramarathon runners. Phillipou et al.^38^ observed a gradual decrease in the levels of circulating sclerostin up to 48 h after a single bout of eccentric exercise, followed by a slight increase at 120 h post-exercise. On the other hand, a study carried out by Kouvelioti et al.^39^ on young men reported an increase in sclerostin 5 min after exercise, similar to that observed after high intensity and high impact exercise (running) or without impact exercise (cycling), and no changes 1, 24, and 48 h post-exercise.

Our findings regarding the levels of sclerostin post-marathon are partly consistent with the results obtained by Kouvelioti et al.^39^, who studied the changes in the sclerostin levels for up to 48 h after exercise. These observations may be related to the intensity of catabolic processes induced by marathon running. However, the level of sclerostin after 72 h was not measured in their study. Thus, we cannot dismiss the possibility that the increased levels of sclerostin in our study, lasting 72 h, are related to catabolic processes induced by strenuous exercise. In fact, this was confirmed by the positive correlation between the changes (Δ_1-2_ and Δ_1-3_) in the sclerostin and hsIL-6 levels observed in our study.

Research has shown that sclerostin acts as a negative regulator of bone mass and strength by inhibiting bone formation^37^. Myostatin has also been described as a negative regulator of bone metabolism, increasing bone resorption by activating the RANKL signaling pathway^8^. Irisin, on the other hand, plays a major role in the regulation of the bone mass, positively influencing cortical mineral density and geometry^14^. However, no association was found between irisin and total body and regional BMD in our study.

It has been reported that osteogenic exercise inhibits sclerostin and myostatin, but induces irisin^40^. However, our research has shown that running a marathon with the differences in altitude along the route induces an upregulation of sclerostin, with a steady increase observed 72 h post-run. Our results suggest that such an intense weight-bearing exercise does not have an osteogenic effect and may lead to disturbances in bone metabolism.

Moreover, the changes in myostatin levels demonstrated in our study suggest a beneficial effect of irisin on myostatin. Recent studies have reported that irisin activates the core-binding factor α-1 and Wnt/β-catenin bone formation pathways^40^, as well as stimulating new bone formation by increasing the osteoblast activity, thus improving bone mass and strength^8^. Thus, the inhibition of myostatin by irisin supports the negative correlation observed between the changes (Δ_1-3_) in the sclerostin and myostatin levels.

Vitamin D plays an important role in bone and muscle metabolism. In our previous study, vitamin D was found to be involved in the inflammatory response^41,42^. In the current study, no significant changes in the serum levels of 25(OH)D were observed post-exercise, and only a slight decrease was found 72 h post-run. Moreover, the positive correlation observed between the changes (Δ_1-3_) in 25(OH)D and irisin suggest that vitamin D has anti-inflammatory properties, confirmed by the results of previous studies^41–45^. Research using animal models demonstrated that a decrease in the levels of 25(OH)D in the blood serum depended on the damage to myocytes and were associated with an increase in the PTH levels^46,47^.

In our study, we observed a significant increase in the levels of PTH 24 and 72 h after the marathon compared to the basal value. These results are consistent previous studies, in which an increase in PTH was reported in the late phase of long-lasting exercise, as well as during recovery^48,49^.

PTH may contribute to skeletal muscle function. Studies on animal models have shown that PTH receptors are expressed on the cell membrane of skeletal muscle fibers, and PTH has been found to modulate the uptake and release of 25(OH)D by muscle cells^50^. No association between irisin or myostatin and PTH was observed in the present study.

PTH stimulates bone turnover. However, depending on other factors, the direction of this stimulus may be towards the formation (anabolic) or resorption (catabolic) of bone. In our study, a negative correlation was observed between the changes (Δ_1-3_) in PTH and OPG, confirming that PTH downregulates OPG expression^51^. The inhibition of OPG expression stimulates osteoclastogenesis and the resorption of osteoclasts, which in turn leads to an increase in the osteoblast pool in the body and stimulates osteoblast activity and bone anabolism^22^.

The marathon race with dominance of eccentric muscle contractions induces mechanical damage to muscle fibers and the corresponding biochemical changes in the blood, as confirmed by previous studies^38,52,53^.

In our study, a significant increase in the levels of myoglobin was observed 24 h post-marathon, followed by a significant decrease after 72 h. However, the levels did not return to the basal value. Myoglobin is released as a result of the degradation of muscle protein structures after strenuous exercise. Its levels can rise within 30 min of the start of exercise, and can remain high for 5 days, most likely due to low-grade inflammation^54^.

We observed a significant increase in the hsIL-6 levels compared to the baseline level 24 h post-marathon, which remained high 72 h after the race. According to Philippou et al.^53^, the increase in IL-6 concentration may indicate its involvement in the acute phase response and inflammatory state regulation. These authors reported a correlation between IL-6 and OPG levels in the blood after high-intensity exercise with a dominant eccentric contraction component. OPG is a key player in suppressing the RANKL/RANK system and may work together with anti-inflammatory cytokines to inhibit inflammation^55^.

In the present study a significant parallel increase in the OPG levels and another classic proinflammatory cytokine, TNFα, were observed 24 and 72 h after the race compared with the baseline level. Some authors have previously suggested that various cytokines (e.g. TNFα) induce the synthesis of OPG by immune cells^56^. These changes are consistent with the results of our previous study, in which we suggested that OPG was involved in inhibiting the inflammatory response^41^. These results are supported by the significant positive correlation observed between the changes (Δ_1-3_) in myoglobin and TNFα and the tendency in the changes (Δ_1-2_) in myoglobin and OPG (r = 0.64, *p* = 0.0537).

Although TNFα is not thought to rise with exercise, its moderate plasma increase may occur^57^. Our results confirm the findings of previous studies, wherein TNFα was stimulated by intense endurance exercise (more than 1 h)^58,59^, suggesting the activation of an inflammatory reaction in response to local damage to active muscles^60^.

CRP plays a role in the induction of anti-inflammatory cytokines from circulating monocytes, and it suppresses the synthesis of pro-inflammatory cytokines from tissue macrophages^61^. We observed a significant increase in the levels of hsCRP 24 h after marathon, followed by a decrease and the tendency to maintain an elevated level during the 72 h recovery period. In contrast, Niemelä et al.^52^ reported a steady increase in the CRP levels even 48 h after exercise. These authors found high levels of CRP, a marker of acute phase response and systemic inflammation, in the subjects with symptoms of post-race fatigue, with the highest values of IL-6 and TNFα^52^.

In our study we did not observe osteopenia in any of the study participants, indicating that they were amateur runners and did not run more than 70 km/week.

In their review, Scofield and Hecht^25^ concluded that, despite weight-bearing exercise being widely recognized as beneficial for long-term bone health, recent research suggests that runners and cyclist often have a lower bone mineral density than athletes participating in power and ball sports. This is most likely due to differences in the degree of physical exertion, such as differences in the loading forces and energy expenditure, during exercise^24,25^. Fredericson et al.^24^ observed a higher BMD in long-distance runners only at directly loaded sites (e.g. calcaneus), but not the rest of the skeleton, in comparison to sedentary men. Additionally, 40% of the runners in their study were found to have lower lumbar spine T-scores consistent with osteopenia, according to the criteria of the World Health Organization (WHO). According to the authors, this may be attributed to increased levels of stress hormones, lower testosterone levels, or increased levels of inflammation markers, but may also be explained by an artificially high reference ranges.

Our study has some potential limitations that are worth noting. The first is the relatively small sample size. The Visegrad Marathon takes place once a year and is popular among recreational runners. Data was obtained from all of the men who responded positively to our invitation to participate in the study, as placed in the race’s announcement. The second limitation is the fact that any changes in biochemical indicators immediately after the marathon race were not assessed, and these could represent a potential narrow window of changes in the measured variables that was missed. Lastly, the mass spectrometry is recognized as a gold standard of determination of serum irisin levels. However, in our laboratory we were able to perform the measurements of irisin levels using the ELISA kit by Aviscera Bioscience with standard range: 0.8–51.2 ng/ml and sensitivity: 100 pg/ml. The producer stated that this ELISA kit was formulated by human irisin derived human cells and monoclonal antibody.

To conclude, our results suggest that in response to a single bout of strenuous exercise lasting more than 3 h, a complex network of endocrine interactions is initiated. Thus, this study provides a basis for further research to fully elucidate the long-term effects of repeated bouts of exercise of high intensity and a long duration.

## Methods

### Subjects

The study group consisted of 10 male was comprised of participants of the Visegrad Marathon race who responded to the invitation to participate in the study. They regularly trained 4–5 times/week, and ran an average of 58.5 km/week.

All study participants (aged 32–51) were characterized by a good state of physical health, recreational level of physical activity, no injuries or chronic conditions, non-smoking, and not taking any medications or dietary supplements that might affect bone health (e.g. vitamin D). The anthropometric, body composition, and bone densitometry characteristics of both groups are shown in Table 2. All of the subjects were informed both verbally and in writing of the experimental protocol prior to signing their written consent to participate. The study protocol was approved by the Ethics Committee for Human Research at the Poznań University of Medical Sciences (reference no. 150/14). All experimental procedures were performed in accordance with the Declaration of Helsinki.

**Table 2:** Baseline characteristics of the study participants.

Variables	Marathon runners (n = 10)
Age (years)	40.6 ± 7.68
Body height (cm)	176.3 ± 4.85
Body mass (kg)	74.7 ± 9.52
Fat (%)	22.1 ± 5.67
Fat mass (kg)	16.1 ± 5.79
Lean mass (kg)	55.6 ± 5.68
Total BMD (g/cm^2^)	1.224 ± 0.090
Total BMD T-score	0.21 ± 0.877
Lumbar spine BMD (g/m^2^)	1.131 ± 0.078
Lumbar spine BMD T-score	− 0.81 ± 0.706
Total femur BMD (g/m^2^)	1.077 ± 0.138
Total femur BMD T-score	− 0.31 ± 0.768
Femoral neck BMD (g/m^2^)	1.014 ± 0.165
VO_2_ max (ml kg^−1^ min^−1^)	48.8 ± 1.83

Data are presented as mean ± SD.

### Marathon race

The running trial took place during the Visegrad Marathon in June (length: 42.195 km, total level difference: 1161 m and total level gain: 491 m). The study participants completed the marathon in times ranging from 3:16:25 to 4:25:20 (mean 3:44:03). The route runs through the Vabec massif in Slovakia and Beskid Sądecki in Poland. The finish line is located in Rytro, at the foot of the Radziejowa Mountain. The route was certified by the Polish Athletics Association (PZLA). On the day of the run, the weather was partly cloudy with a temperature ranging from 10 °C at the start to 23 °C at the finish line, and a relative humidity between 40 and 46%. During the marathon, liberal amounts of fluids and exogenous carbohydrates were available to the runners.

### Anthropometric, body composition, and bone densitometry measurements

Body mass and height were measured using a certified digital medical scale (model WPT 80/150.O; Radwag, Radom, Poland), with an accuracy of 0.01 kg, and a mechanical measuring rod to measure body height, with an accuracy of 0.5 cm.

Dual-energy X-ray absorptiometry (DXA) body composition and BMD measurements were performed on an empty stomach using a GE Lunar Prodigy Primo Full Densitometer with the enCore Body Composition option (GE Healthcare Technologies, USA). The assessment of BMD were acquired for the total body, lumbar spine (L1–L4) and left hip (total femur and femoral neck). All measurements were performed one week before the start of the marathon.

### Physical fitness measurement

Two weeks before the start of the marathon, the study participants performed a treadmill exercise test (HP Cosmos Saturn, Germany), as described previously^62^. The test was initiated at a baseline speed of 6 km/h and then continuously increased. At 3-min intervals, the speed of the treadmill was increased by 2 km/h, up to the maximal speed for a given subject characterized by the lack of increase in minute oxygen uptake despite increased exercise intensity. The test was not preceded by a warm-up session. The cardiovascular and respiratory parameters were monitored continuously using an ergospirometer (VO_2_ max Finder; MES, Poland). The heart rate was recorded every 5 s using a Polar Accurex Plus device (Polar Elektro, Finland).

### Biochemical analyses

Three samples of blood in fasting conditions (between 08:00 AM and 10:00 AM), were obtained from the antecubital vein for biochemical analyses (1) 24 h before the marathon, (2) 24 h after the marathon, (3) 72 h after marathon. The samples were collected with all safety standards into serum-separating tubes (9 ml, S-Monovette, SARSTEDT, Nümbrecht, Germany) and centrifuged at 2000 × g for 10 min at 4 °C. The serum was separated from the sample and stored at − 70 °C.

Circulating levels of myoglobin, hsCRP, and PTH levels were determined by immunoenzymatic assay using commercially available kits (DRG International Inc., Springfield Township, NJ, USA; test sensitivity: 5 ng/mL, 0.1 mg/L, and 1.57 pg/mL, respectively; intra-assay coefficients of variations (CV): 5.4%, 4.4%, 4.9%, respectively; inter-assay CV: 8.3%, 3.3%, 3.2%, respectively), as well as the levels of hsIL-6 and TNFα (R&D Systems Inc., Minneapolis, MN, USA; test sensitivity: 0.039 pg/mL and 5.5 pg/mL, respectively; intra-assay CV: 4.1%, 4.7%, respectively; inter-assay CV: 6.5%, 5.8%, respectively), irisin (Aviscera Bioscience Inc., Santa Clara, CA, USA; test sensitivity: 100 pg/mL; intra-assay CV: 4.2%; inter-assay CV: 5.6%), myostatin (Immundiagostic AG, Bensheim, Germany; test sensitivity: 0.37 ng/mL; intra-assay CV: 9.1%; inter-assay CV: 13.1%), osteoprotegerin (OPG) and sclerostin (Biomedica GmbH & Co KG, Wien, Austria; test sensitivity: 0.07 pmol/L and 3.2 pmol/L, respectively intra-assay precision CV: 2.5%, 6.0%, respectively; inter-assay precision CV: 4.0%, 6.5%, respectively). The serum concentration of 25(OH)D was determined by chemiluminescent immunoassay (CLIA, DiaSorin Liaison, Stillwater, USA; test sensitivity: 4 ng/mL intra-assay precision CV: 5.4%; inter-assay precision CV: 4.9%).

### Statistical analysis

The data are presented as the mean ± standard deviation (SD). The Shapiro–Wilk test was used to check the normality of the data distribution. The assumption on sphericity was tested using Mauchley’s test (verifying if variances of certain variables were identical and equal to respective co-variances). One-way analysis of variance (ANOVA) with repeated measures was used to compare one quantitative variable with a normal distribution at three points in time. When ANOVA showed significance, the post-hoc Tukey’s honestly significant different test was used to indicate the measurements tested in three time points of the study that were significantly different. For data that was not normally distributed, Friedman’s non-parametric test was used for the comparison of repeated measured values over the study period at the three time points, followed by the Dunn’s post-hoc test to detect differences between each time point. The statistical significance was set at *p* < 0.05. Relationships between variables were tested using Spearman’s rank correlation. All analyses were performed using the Statistica 13.3 software package (TIBCO Software Inc., USA).

## Supplementary information

Supplementary information.

Supplementary figure legend.

Supplementary figure S1.

Supplementary table S1.

## Data Availability

The datasets generated during and/or analyzed during the current study are available from the corresponding author on reasonable request.
