# Supplementary material for: Effects of marathon race on selected myokines and sclerostin in middle-aged male amateur runners
Source: Sci Rep. 2021 Feb 2;11:2813. doi: 10.1038/s41598-021-82288-z (PMC7854637; doi:10.1038/s41598-021-82288-z)
Supplement: Supplementary file 2 — Supplementary figure legend. [file 41598_2021_82288_MOESM2_ESM.pdf]

Effects of marathon race on selected myokines and sclerostin in middle-aged male amateur runners

Figure S1. The route of the Visegrad Marathon
