# Supplementary material for: Effects of marathon race on selected myokines and sclerostin in middle-aged male amateur runners
Source: Sci Rep. 2021 Feb 2;11:2813. doi: 10.1038/s41598-021-82288-z (PMC7854637; doi:10.1038/s41598-021-82288-z)
Supplement: Supplementary file 4 — Supplementary table S1. [file 41598_2021_82288_MOESM4_ESM.pdf]

Effects of marathon race on selected myokines and sclerostin in middle-aged male amateur runners

Table S1. Blood concentrations of biochemical indices in marathon runners

	Before (1)	*p-value 1 vs 2*	24 h after (2)	*p-value 2 vs 3*	72 h after (3)	*p*-value 1 *vs* 3	*ANOVA p-value*	Effect size η^2
Myostatin [ng/mL]	24.5 ± 4.10	0.0002	30.29 ± 5.34	0.9400	30.0 ± 4.82	0.0002	<0.0001	0.72
Irisin [ng/mL]	7.3 ± 0.98	0.1771	7.9 ± 1.33	0.4443	8.3 ± 0.98	0.0159	0.0200	0.35
Sclerostin [pmol/mL]	25.8 ± 5.67	0.1113	30.0 ± 6.46	0.2831	33.0 ± 6.24	0.0045	0.0059	0.43
OPG [pmol/mL]	3.3 ± 0.88	0.0002	4.8 ± 1.29	0.0387	4.2 ± 1.46	0.0069	0.0000	0.68
25(OH)D [ng/mL]	30.3 ± 4.52	0.9896	30.4 ± 5.04	0.0744	28.7 ± 6.24	0.0964	0.0529	0.23
PTH [pg/mL]	99.9 ± 38.62	0.0112	130.7 ± 10.97	0.7133	138.1 ± 18.79	0.0021	0.0017	0.51
IL-6 [pg/ml]	5.2 ± 2.77	0.0001	9.7 ± 4.61	0.0019	7.2 ± 3.62	0.0114	<0.0001	0.75
TNFα [pg/mL]	2.4 ± 0.61	0.0001	6.2 ± 1.85	0.0002	4.6 ± 1.56	0.0069	0.0000	0.83
hsCRP [mg/L]	0.02 ± 0.015	0.0001	0.10 ± 0.036	0.0002	0.05 ± 0.026	0.0510	<0.0001	0.79
Myoglobin [ng/mL]	49.0 ± 18.35	0.0001	199.5 ± 95.92	0.0304	97.3 ± 53.30	0.3526	0.0001	0.69

Data are presented as mean ± SD; 1: before marathon race, 2: 24 h after marathon race, 3: 72 h after marathon race
